# FOS as a biomarker for myocardial infarction treatment with Deng's Yangxin Decoction: a systems biology-based analysis

**DOI:** 10.3389/fcvm.2025.1488684

**Published:** 2025-05-30

**Authors:** Junfeng Fang, Wei Wu, Weifeng He, Lin Wang, Shiyi Liu

**Affiliations:** ^1^ Department of Emergency Medicine, First Affiliated Hospital of Guangzhou University of Chinese Medicine, Guangzhou, China; ^2^ Department of Cardiovascular Medicine, First Affiliated Hospital of Guangzhou University of Chinese Medicine, Guangzhou, China; ^3^ The First Clinical Medical College, Guangzhou University of Chinese Medicine, Guangzhou, China

**Keywords:** myocardial infarction, immune infiltration, bioinformatic analysis, single-cell analysis, network pharmacology, diagnostic biomarker

## Abstract

**Background:**

Deng's Yangxin Decoction (DYX) is a Chinese herbal formula used in clinical practice to treat patients with myocardial infarction (MI). However, its underlying mechanism remains unclear.

**Objective:**

This study aims to explore potential biomarkers and associated mechanisms of DYX for MI.

**Methods:**

Therapeutic targets for DYX were obtained based on the Traditional Chinese Medicine Systems Pharmacology Database and Analysis Platform, Traditional Chinese Medicine Integrated Database, and UniProt databases. Key targets were screened using topological analysis. Differentially expressed genes (DEGs) between MI patients and controls were obtained using open-source datasets. Weighted gene co-expression network analysis (WGCNA) was utilized to screen MI-related genes in the expression array. Hub biomarkers were determined by intersecting DEGs, protein–protein interaction networks, and WGCNA results. Molecular docking validated interactions between DYX components and hub biomarkers. Immune infiltration was assessed via CIBERSORT. Single-cell RNA sequencing analyzed hub biomarker expression in coronary plaques.

**Results:**

FOS was a core biomarker for DYX for MI. Molecular docking confirmed strong binding affinities between quercetin/baicalein and FOS. In addition, high expression of FOS was associated with immune infiltration of neutrophils, activated mast cells, activated dendritic cells, monocytes, and NK cells. FOS was also found to be expressed at high levels in mast and dendritic cells, monocytes, and some T cells in coronary plaques.

**Conclusion:**

FOS is a target of DYX for the treatment of MI, and the mechanism of action may be related to the modulation of immune infiltration.

## 1 Introduction

Myocardial infarction (MI), a critical manifestation of atherosclerotic cardiovascular disease (ASCVD), results from persistent coronary ischemia leading to myocardial necrosis and subsequent complications including heart failure and arrhythmias (1–3). Despite advances in cardiovascular care, ASCVD continues to claim more lives globally than any other disease, a trend projected to persist for decades (4). The early stage of acute MI (AMI) carries the highest risk of sudden death, malignant arrhythmias, acute heart failure, and cardiogenic shock (5, 6). These observations underscore the vital need for early detection and intervention.

Current diagnostic paradigms rely heavily on serum biomarkers such as troponin, yet their delayed elevation and limited specificity hinder timely diagnosis (7). Therapeutic strategies face dual challenges: While revascularization remains the cornerstone treatment, it demonstrates limited efficacy in patients with advanced ventricular dysfunction (8). Pharmacotherapies including antiplatelet agents and β-blockers, though life-saving, carry inherent bleeding risks that complicate long-term management (9, 10). Emerging epigenetic biomarkers may address these diagnostic gaps. Circulating microRNAs (miRNAs), such as miR-1, miR-133a, and miR-208 families, exhibit rapid release kinetics (detectable within 1–3 h post-MI) and tissue-specific expression patterns, offering advantages over conventional troponin assays in early diagnosis and infarct localization (11–13). Notably, miR-499 achieves 90.6% accuracy in differentiating MI from other cardiac injuries (13). However, standardization challenges in detection platforms and biological variability currently limit clinical translation (14).

Beyond diagnostic innovation, therapeutic strategies capable of modulating both ischemic injury and its molecular triggers are urgently needed. This dual requirement positions traditional formulations such as Deng's Yangxin Decoction (DYX) as compelling candidates. DYX, a traditional Chinese medicine formula developed by Professor Tietao Deng, is commonly used for post-MI care based on Chinese medicine principles and shows certain therapeutic effects (15, 16). Composed of *Panax ginseng*, *Ophiopogon japonicus*, *Pinellia ternata*, and *Panax notoginseng*, DYX exhibits multimodal cardioprotective properties. A meta-analysis demonstrated that *Panax ginseng* inhibits atherosclerosis by reducing levels of proinflammatory cytokines TNF-α and IL-6 (17). Another meta-analysis revealed that when combined with routine therapy, *Panax notoginseng* saponins injection can reduce infarct size and inflammation in AMI patients, improving treatment outcomes (18). *Panax ginseng* saponin has been reported to reduce myocardial ischemic damage by enhancing glucose deprivation-induced autophagy (19). Ginsenoside Rg1 inhibits the activation of the AIM2 inflammasome, thereby alleviating the inflammatory response and myocardial fibrosis following MI (20). Ophiopogonis polysaccharide can promote cardiac microangiogenesis after myocardial ischemia, thus protecting cardiomyocytes (21). Ophiopogonin D can reverse palmitic acid-induced lipid accumulation and mitochondrial damage, thus protecting cardiomyocytes (22). An animal experiment showed that notoginsenoside R1 inhibits the phosphorylation of transforming growth factor β-activated kinase 1 (TAK1), thereby inhibiting the JNK/p38 pathway to reduce myocardial ischemia–reperfusion injury (23). Despite these empirical observations, DYX's mechanistic underpinnings in MI management remain poorly characterized.

Recent immunological insights reveal that post-MI cardiac repair involves dynamic immune cell interactions, particularly macrophage polarization (24–26). The infarct microenvironment drives macrophage differentiation toward M1 phenotypes through surface receptors such as Dectin-1 and CXCR7 (27–29). These proinflammatory M1 macrophages play a transient protective role by engulfing cellular debris and degrading damaged extracellular matrix via MMP-9 secretion (30). However, persistent M1 dominance creates a self-perpetuating inflammatory cycle—excessive TNF-α/IL-1β production recruits additional monocytes while suppressing anti-inflammatory TGF-β signaling, ultimately impairing scar maturation and promoting fibrotic overgrowth (31). In contrast, M2 macrophages differentiated from Ly6C(low) monocytes begin to exert anti-inflammatory, angiogenic, and cardioprotective effects (32). This paradoxical transition from essential repair to pathological remodeling highlights the critical need for balanced immune regulation, a process potentially modulated by DYX's bioactive components (33–36). This positions immune cell modulation as a plausible therapeutic axis for DYX's observed clinical benefits.

**Figure 1:**
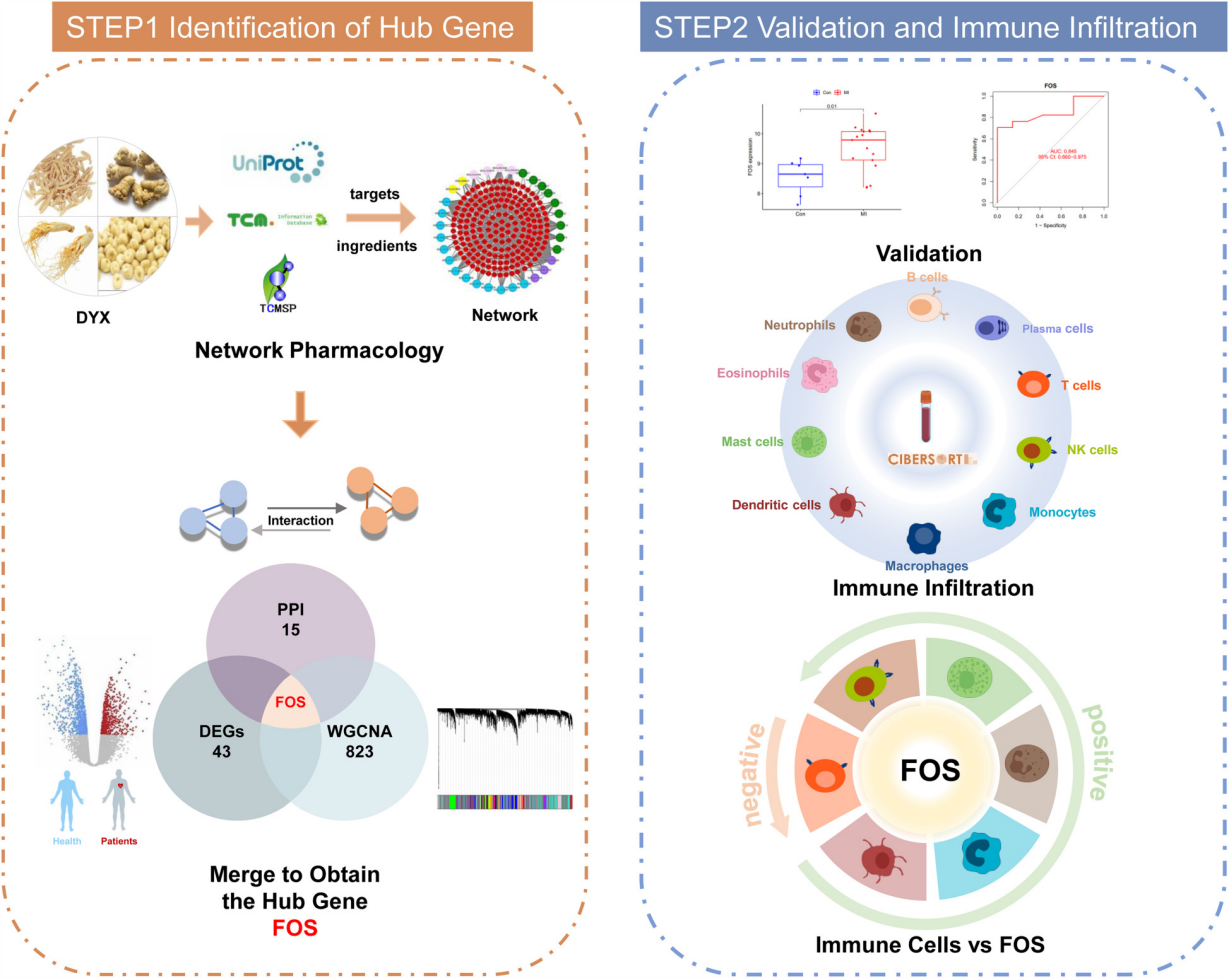
Workflow of the systematic strategies to elucidate the mechanisms of the treatment of DYX on MI. DYX, Deng's Yangxin Decoction; MI, myocardial infarction.

To bridge these knowledge gaps, we employed an integrative strategy combining network pharmacology, bioinformatics, and machine learning. We identified DYX's putative targets through compound-target network analysis. Transcriptomic profiling revealed MI-associated differentially expressed genes (DEGs). Machine learning was employed to screen and validate the DEGs. Combining the three analyses, potential signature genes of DYX for MI were obtained. Strong binding affinities between core components and key targets were validated with molecular docking. In addition, immune infiltration patterns were quantitatively assessed to delineate cellular subpopulation dynamics. Finally, correlation analyses linked signature genes with specific immune subsets, elucidating potential mechanisms of DYX-mediated cardioprotection. The workflow of the study is shown in Figure 1.

## 2 Materials and methods

### 2.1 Construction of protein–protein interaction (PPI) network

The active ingredients of four herbs in DYX were obtained from Traditional Chinese Medicine Systems Pharmacology Database and Analysis Platform (TCMSP; developed by College of Life Science, Northwest A&F University, available at https://tcmspw.com/tcmsp.php) and Traditional Chinese Medicine Integrated Database (TCMID; developed by Shanghai Key Laboratory of Regulatory Biology, accessible at https://megabionet.org/tcmid/) using oral bioavailability (OB) ≥30% and drug-likeness (DL) ≥ 0.18 as screening criteria (37, 38). The targets of active ingredients in DYX were obtained from the TCMSP database. The protein sequences of the targets were converted into official gene symbols by the UniProt database (a comprehensive resource maintained by the European Bioinformatics Institute, the Swiss Institute of Bioinformatics, and the Protein Information Resource, available at https://www.uniprot.org/). The STRING (developed by the European Molecular Biology Laboratory, accessible at https://www.string-db.org/) database was applied to analyze the interaction between active ingredients and targets. The Cytoscape software was used to construct the “active ingredient–target” network. The protein interactions were searched by the SRTING database. The targets of DYX were entered into the database, and the scoring condition was set to >0.40 to obtain the protein–protein interaction (PPI) network of DYX. The network was visualized and topologically analyzed in Cytoscape, and nodes with a degree higher than the median were filtered multiple times to obtain core targets.

### 2.2 Data collection

Datasets were derived from the Gene Expression Omnibus (GEO) database (https://www.ncbi.nlm.nih.gov/geo/) with the following keywords: (Myocardial Infarction) AND “Homo sapiens” AND “Expression profiling by array” AND “Series”. The screening standards included the following: the microarray datasets referred to profiles of gene expressions of whole blood; the microarray datasets contained samples from MI and samples from a healthy state; all included samples were not treated with drugs. Eventually, datasets GSE66360 (39) and GSE60993 (40) were selected for the next analysis. The clinical information for the samples of MI patients and healthy controls was provided in Supplementary Table S1.

### 2.3 Differential expression analysis

The LIMMA package in R software was utilized to identify differentially expressed genes (DEGs) through the comparison of the expression dataset in GSE66360. The volcano plot and heatmap were drawn to present the expression of DEGs. DEGs with adjusted *p* < 0.05 and |log_2_FC| > 2 were considered statistically significant.

### 2.4 Functional enrichment analysis

For the exploration of the function and pathway of the DEGs, our study conducted the Metascape (http://metascape.org/gp/index.html) analysis, choosing species as “*H. sapiens*.” Meanwhile, gene ontology (GO) enrichment analysis was performed with the use of the clusterProfiler package, and adjusted *p* < 0.05 was considered statistically significant. Disease ontology (DO) analysis in the clusterProfiler package was also employed to analyze the relationship between disease and DEGs. To more intuitively clarify the gene expression level of significantly enriched functional pathways, gene set enrichment analysis (GSEA) was performed in R software.

### 2.5 Construction of weighted gene co-expression network

After excluding batch effects, median absolute deviation (MAD) was calculated for gene expression, and the top 10% of genes with the smallest MAD values were removed. Outlier genes and samples were culled using the WGCNA package in R software, and a scale-free co-expression network was constructed by soft thresholding. The optimal soft threshold was chosen to cluster this network into different functional modules, each containing a minimum of 30 genes. Modules were hierarchically clustered by calculating the eigengene, and similar modules were merged. Pearson’s correlation analysis was used to determine the correlation between modules and clinical features by combining gene significance (GS) and module membership (MM). Clinical modules with high correlation with MI samples and normal samples were selected, and the core genes were finally screened out from the target modules.

### 2.6 Identification and verification of hub biomarkers

In this study, a Venn diagram was used to obtain the intersection of DEGs, core targets in the PPI network, and core genes in the WGCNA for further analysis.

For the in-depth test of the efficacy of key biomarkers, the GSE60993 dataset served as the validation set. It was assessed based on the receiver operating characteristic (ROC) with the pROC package, and the area under the curve (AUC) was calculated to evaluate the predictive effect achieved by the model. A two-sided *p* < 0.05 showed statistical significance. Meanwhile, the different expression level of candidate genes between healthy people and MI patients was presented with the ggpubr package, *p* < 0.05 showed statistical significance.

### 2.7 Molecular docking validation

To predict the binding of candidate gene proteins to small molecules of components of DYX, we performed molecular docking after optimizing the molecular structure and the free energy of small ligand molecules and acceptor proteins. First, the PDB files of the target protein were downloaded from the PDB database (http://www.rcsb.org/). The proteins were saved in PDBQT format as the docking acceptor after dehydration and hydrogenation. Next, the small-molecule 2D structures were downloaded from the PubChem database (https://pubchem.ncbi.nlm.nih.gov/). The minimum free energy was calculated, and the structure was optimized and saved as a mol2 format file. Finally, molecular docking was performed using AutoDock Vina to estimate the binding activity of small molecules to proteins mainly by the binding free energy. The lower the binding energy value, the greater the likelihood that the ligand will bind to the receptor.

### 2.8 Evaluation and correlation analysis of infiltration-related immune cells

The CIBERSORT algorithm was adopted to identify 22 immune cells in the samples. CIBERSORT *p* < 0.05 showed statistical significance. The reshape2, ggpubr, and ggExtra packages were applied to demonstrate the different distributions of immune cells in healthy controls and patients with MI. Based on Spearman correlation analysis, correlations were calculated for 22 immune cells. The results of the correlations were visualized by a heatmap using the corrplot package. The difference between groups of 22 immune cells was analyzed based on the *t*-test, and adjusted *p* < 0.05 was statistically different. The results were visualized using the ggpubr package.

The correlation of hub genes with 22 immune cells was determined using the psych package. Results were visualized using the ggpubr and reshape2 packages.

### 2.9 Processing of single-cell dataset

A single-cell RNA sequencing (scRNA-seq) dataset [GSE184073 (41)] of immune cells in coronary plaques was obtained from the Gene Expression Omnibus (GEO) database (https://www.ncbi.nlm.nih.gov/geo/). Two coronary plaque samples were included in the dataset, one from a patient with acute coronary syndrome (ACS) and the other from a patient with stable angina pectoris (SAP).

The “Seurat” (42) package in R software was applied to read the dataset. Low-quality cells were excluded using the following criteria: (1) features < 200 and >5,000 and (2) mitochondrial genes >25%. First, the expression data of the screened cells were normalized. Then, 2,000 highly variable genes were obtained for principal component analysis (PCA). After integrating the two samples using the “harmony” (43) package to remove batch effects, the first 30 principal components in PCA were used for subsequent cell clustering. Finally, dimensionality reduction was performed using the uniform manifold approximation and projection (UMAP) algorithm.

The “FindAllMarkers” function was employed to identify DEGs between cell clusters as markers of cells. CellMarker (http://bio-bigdata.hrbmu.edu.cn/CellMarker) (44) is a database that collects cell markers identified in previous articles. Cell clusters were manually annotated by reference to CellMarker.

### 2.10 Exploring FOS expression in cell clusters

The “AddModuleScore” function was utilized to assess the expression of FOS in different cell clusters. Violin plots and feature plots were used to demonstrate FOS expression in different samples or different cell clusters.

FOS expression was not consistent across T cells. All T cells were categorized into a high FOS group and a low FOS group. The “FindMakers” function was used to identify DEGs between the two groups (adjusted *p* < 0.05 and |log_2_FC| > 0.5). To further explore the differences in the involvement of T cells in biological processes, Kyoto Encyclopedia of Genes and Genomes (KEGG) enrichment analysis was performed on DEGs.

## 3 Results

### 3.1 Analysis results of PPI networks

**Figure 2:**
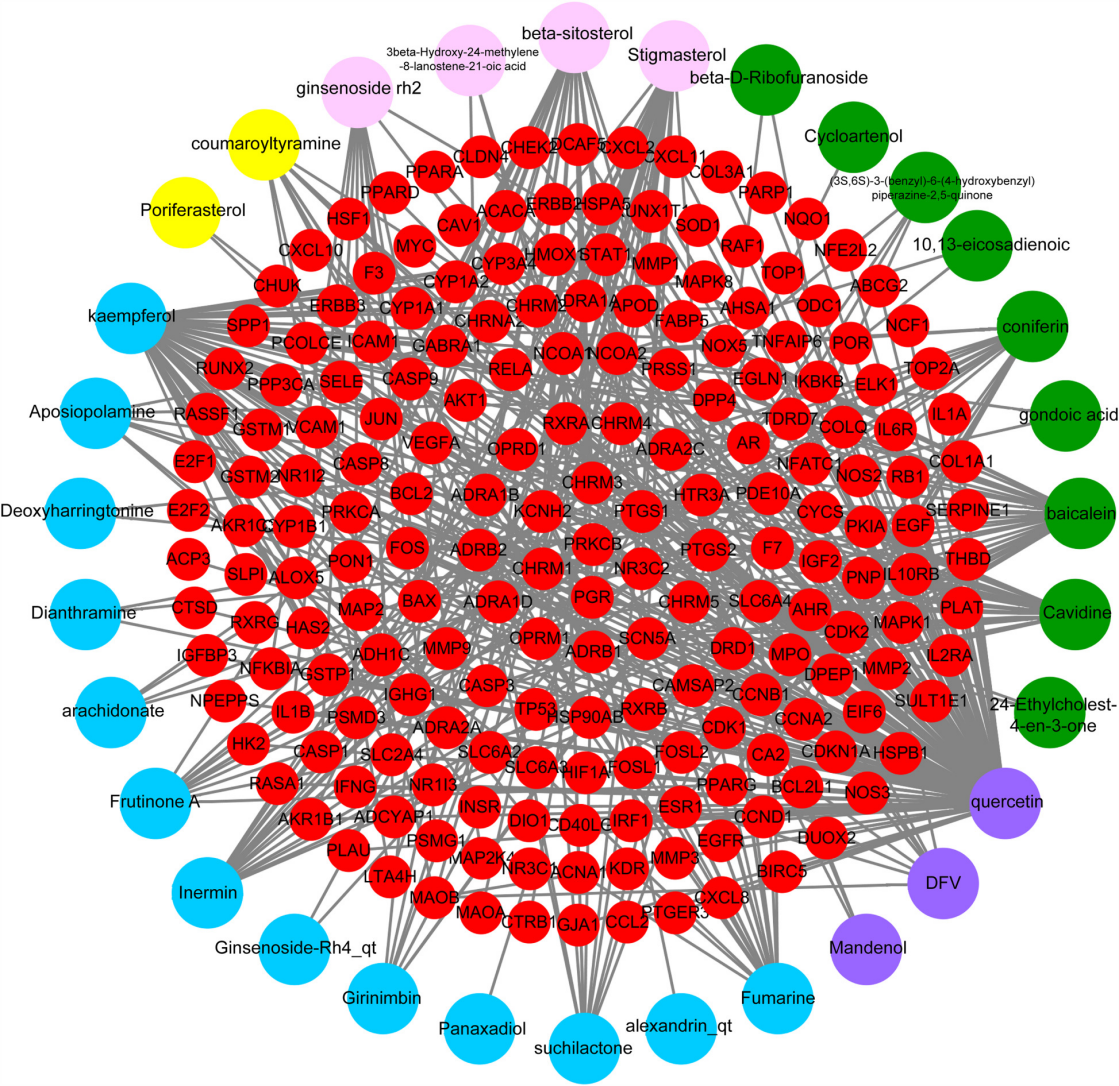
Ingredient–target network of DYX. The network of the relationship between the active ingredients and the targets. PPI, protein–protein interaction; DYX, Deng's Yangxin Decoction.

**Figure 3:**
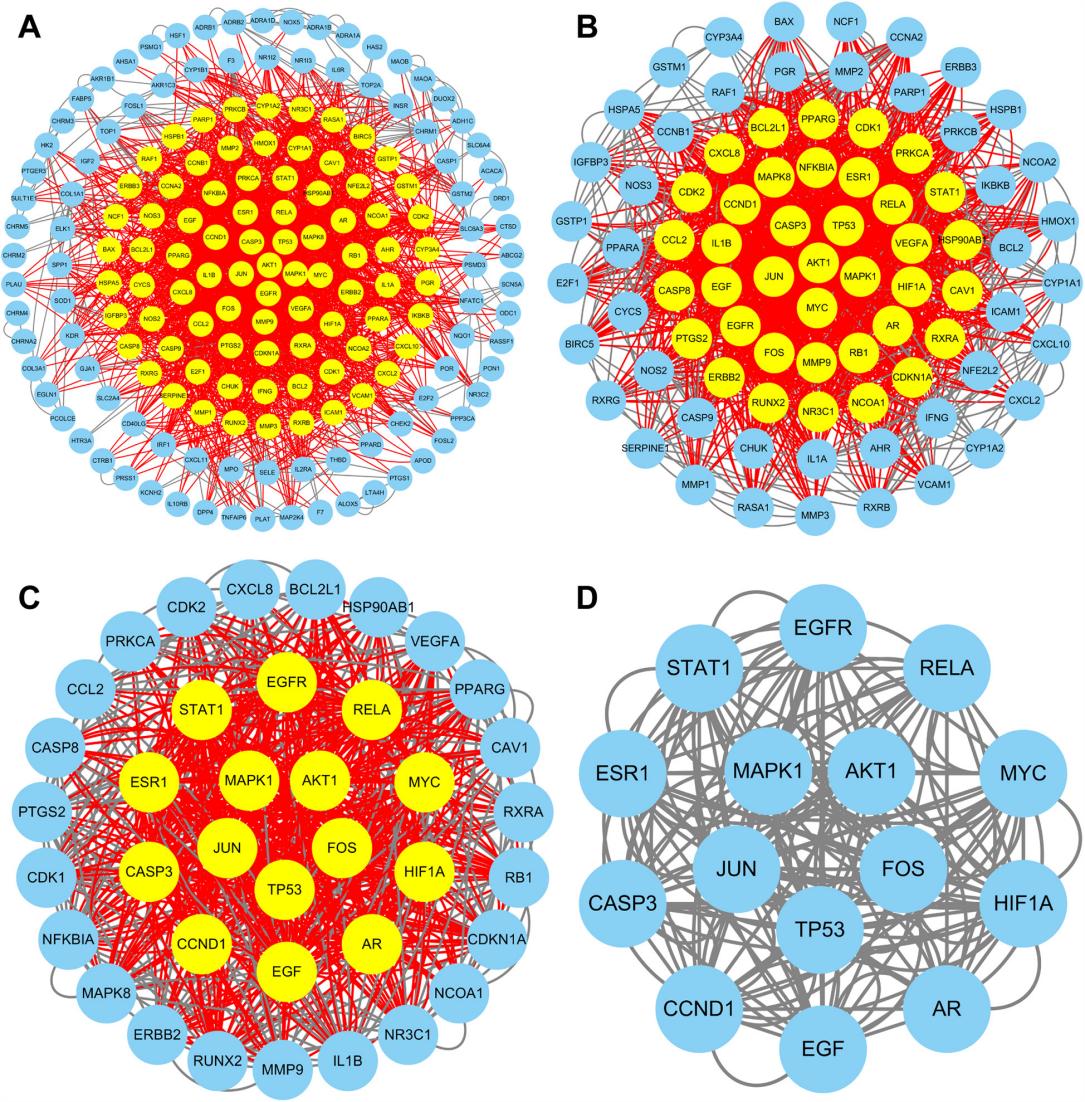
PPI network of targets. Targets are shown with yellow dots (high degree) and blue dots (low degree). PPI networks of all targets **(A)**, targets with a degree value of >8 **(B)**, and targets with a degree value of >15 **(C)**. **(D)** PPI network of 15 core targets (degree > 17).

The TCMSP database and TCMID were searched for the active ingredients of DYX, and a total of 31 active ingredients were obtained after screening. A total of 198 non-duplicated targets were obtained by searching using these active ingredients individually. Figure 2 illustrates the “active ingredient–target” network of DYX. The outermost dots are the active ingredients of DYX, and the inner red dots are the targets. After removing the 26 non-interacting targets, the remaining 172 targets constituted the PPI network (Figure 3A). There are 172 nodes and 1,042 edges in the network. The core targets were then screened multiple times using the median of the degree. Firstly, targets with a degree value of >8 were screened, leaving 82 nodes and 717 edges (Figure 3B); next, targets with a degree value of >15 were screened, with 38 nodes and 313 edges remaining (Figure 3C); and finally, targets with a degree value of >17 were screened, resulting in a network consisting of 15 nodes and 90 edges (Figure 3D). A total of 15 selected core potential therapeutic targets were obtained: FOS, STAT1, EGFR, RELA, MYC, HIF1A, AR, EGF, CCND1, CASP3, ESR1, MAPK1, AKT1, JUN, and TP53.

### 3.2 DEGs and functional enrichment analysis

**Figure 4:**
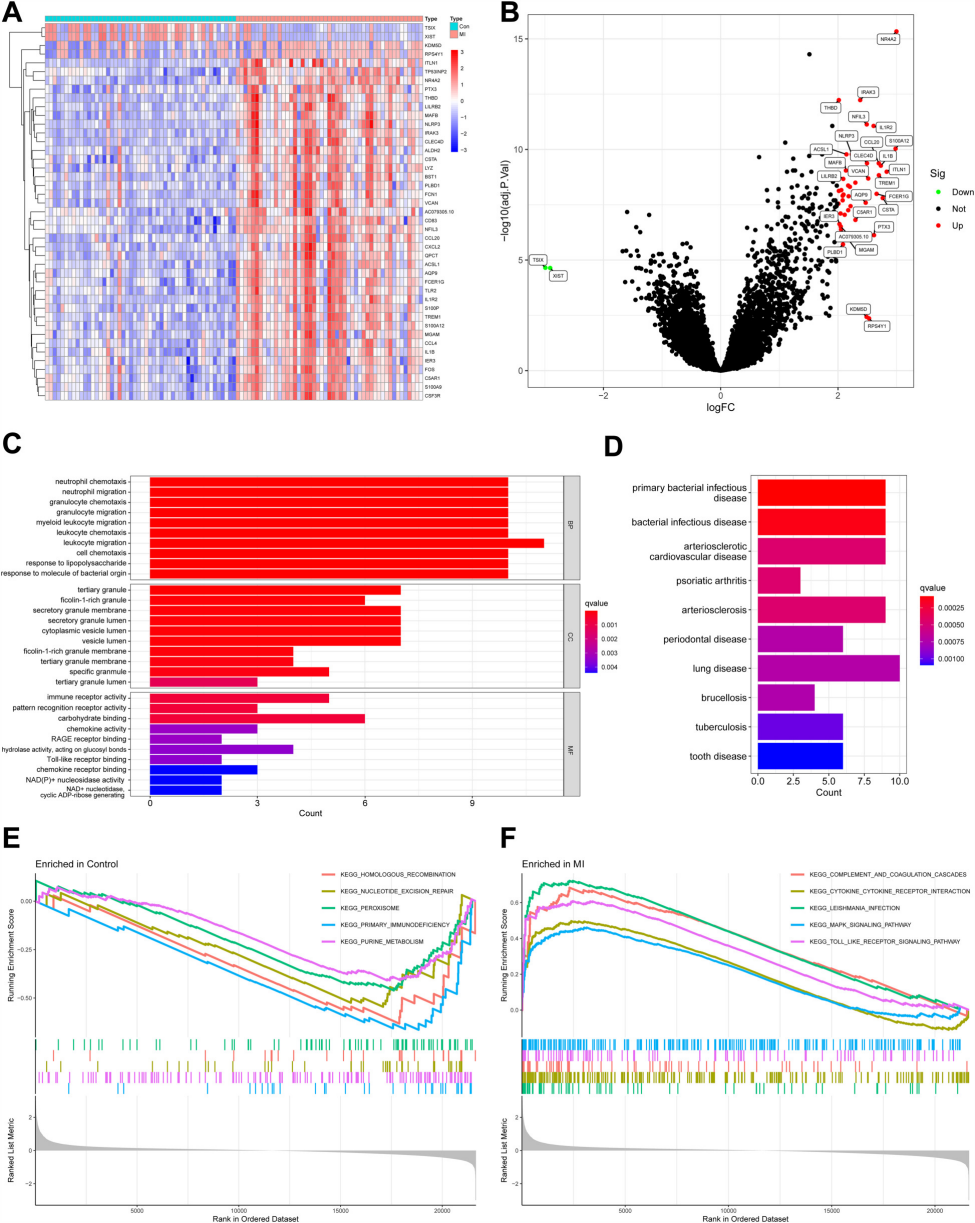
DEGs between healthy people and MI patients and their functional enrichment analysis. **(A)** Heatmap of DEGs. Red indicates relatively high expression of this gene in the sample, and blue indicates relatively low expression. **(B)** Volcano plot of DEGs. Red dots represent genes significantly upregulated in MI samples, and green dots represent genes significantly downregulated. **(C)** GO enrichment analysis of DEGs. Bar length reflects the number of genes associated with each term (count), while color intensity represents statistical significance (*q*-value, red = more significant). **(D)** DO analysis of DEGs. Diseases are ranked by significance (*q*-value, red = most significant), and bar length indicates the number of genes linked to each disease (Count). **(E)** GSEA comparing gene expression profiles between control and MI samples, highlighting pathways enriched in the control group. **(F)** GSEA highlighting pathways enriched in MI samples compared with controls. DEGs, differentially expressed genes; MI, myocardial infarction; GO, gene ontology; DO, disease ontology; GSEA, gene set enrichment analysis.

A total of 43 DEGs were identified between healthy people and patients with MI in GSE66360, and the heatmap shows the expression of 43 DEGs between the two groups (Figure 4A). As shown in Figure 4B, red dots indicated upregulated genes (41 genes), green dots indicated downregulated genes (2 genes), and black dots indicated no statistical significance. The expression of most DEGs was downregulated in healthy individuals and upregulated in patients with MI.

GO enrichment analysis (Figure 4C) showed that DEGs were associated with migration of neutrophils and myeloid leukocytes, suggesting immune cell infiltration after MI. Tertiary granules, a structure of leukocytes, were also shown to be associated with DEGs. The molecular function section showed that these DEGs were also involved in immune receptor activity and pattern recognition receptor activity. DO analysis indicated that DEGs were associated with arteriosclerosis and bacterial infectious diseases (Figure 4D). This may be associated with the involvement of these genes in plaque formation and the promotion of inflammation.

GSEA revealed distinct pathway enrichments in healthy individuals and MI patients. In controls, downregulated DEGs were linked to “primary immunodeficiency,” “purine metabolism,” “homologous recombination,” “nucleotide excision repair,” and “peroxisomal enzymes” (Figure 4E). These pathways are crucial for immune regulation and cellular homeostasis, and their inhibition suggests a stable immune state in healthy individuals. In MI patients, upregulated DEGs were associated with “complement and coagulation cascades,” “cytokine–cytokine receptor interactions,” “leishmania infection,” “MAPK signaling pathway,” and “Toll-like receptor signaling pathway” (Figure 4F). The “complement and coagulation cascades” pathway indicates an immune response to tissue damage, while “cytokine–cytokine receptor interactions” suggests amplified inflammatory signaling. The “MAPK” and “Toll-like receptor signaling pathways” are vital for initiating immune and inflammatory responses. These findings imply that post-MI, immune cells, particularly leukocytes, infiltrate the affected area as part of the inflammatory response to myocardial damage. This analysis underscores the immune system's significant involvement in MI's pathophysiology.

### 3.3 Weighted gene co-expression networks

**Figure 5:**
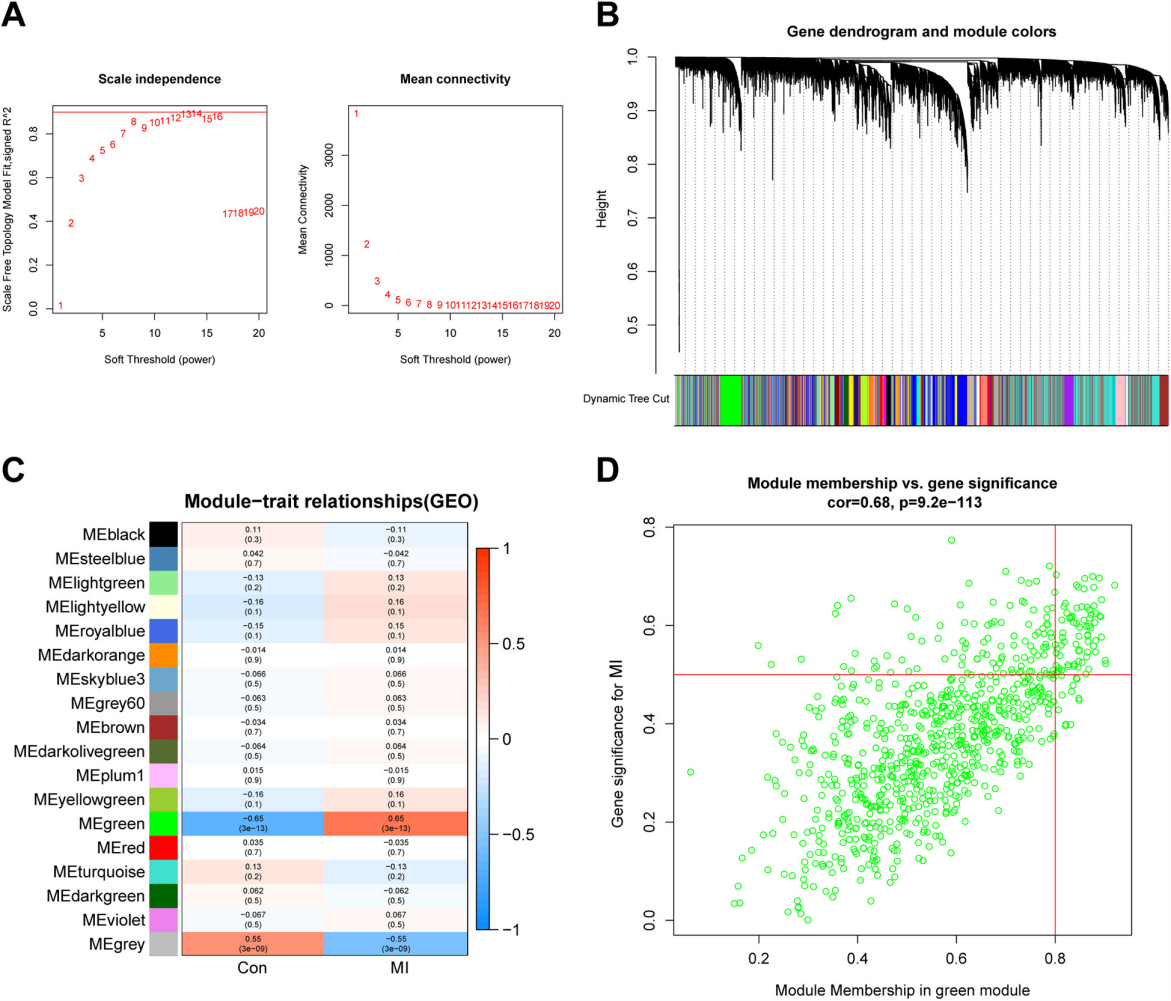
WGCNA. **(A,B)** Analysis of the scale-free index and the mean connectivity for various soft-thresholding powers. **(C)** Relationships of consensus modules with samples. Each specified color represents a specific gene module. **(D)** The gene significance for MI in the green module. WGCNA, weighted gene co-expression network analysis; MI, myocardial infarction.

In this study, we set the soft threshold to 13 (*R*^2^ = 0.98) (Figure 5A). Then, 18 modules were obtained based on average hierarchical clustering and dynamic tree shearing (Figure 5B). Among them, the green module was highly correlated with MI and was therefore selected for further analysis (Figure 5C). Correlation analysis between MM in the green module and gene significance for MI also showed that the green module containing 828 genes was relevant to MI (Figure 5D, *r* = 0.68, *p* = 9.2 × 10^−^¹¹³).

### 3.4 Hub biomarker identification and verification

**Figure 6:**
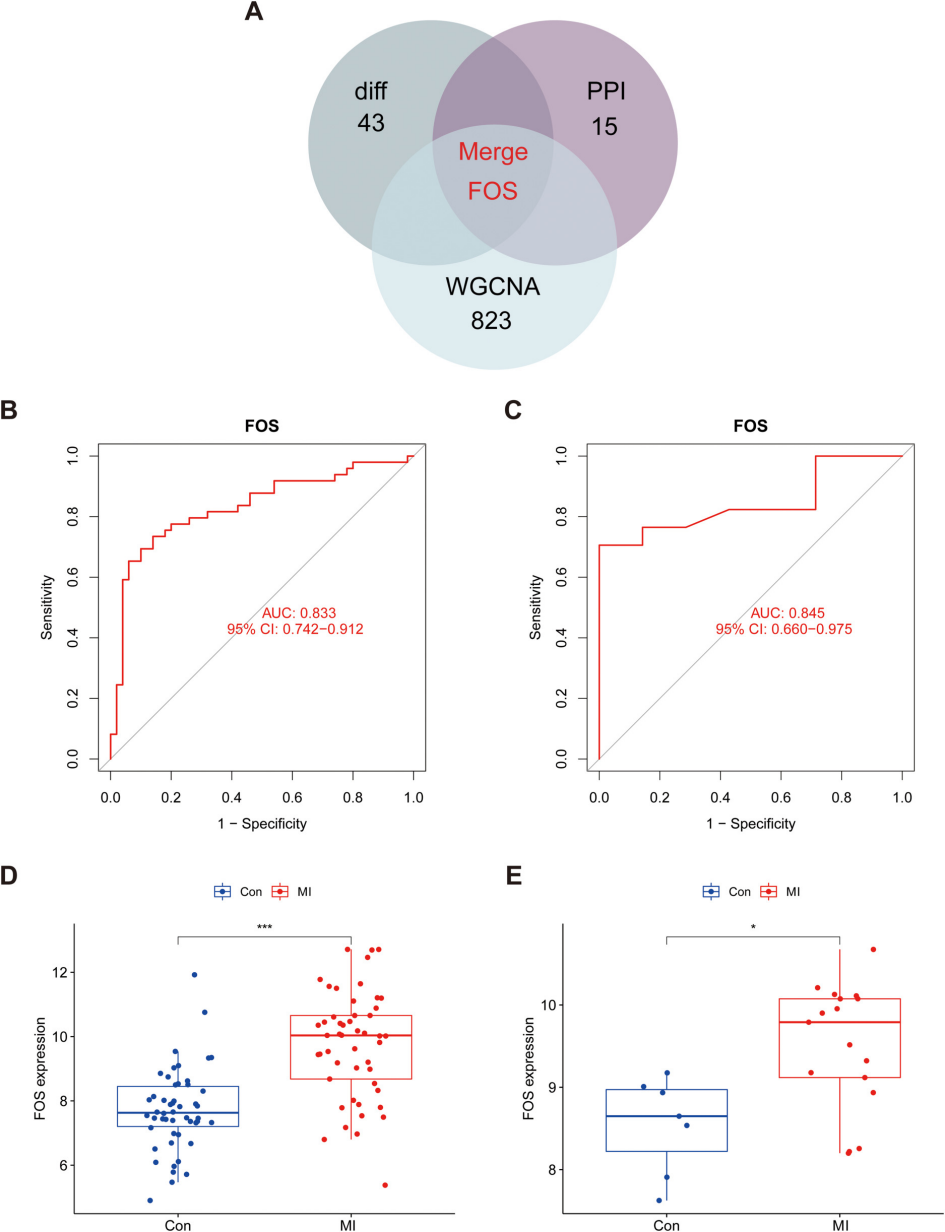
Identification and validation of hub marker for MI. **(A)** Venn diagram showing the intersection of DEGs, core targets in the PPI network, and hub genes in WGCNA. **(B)** The ROC curve of the diagnostic characteristic marker in GSE66360. **(C)** The ROC curve of the diagnostic characteristic marker in GSE60993. **(D)** FOS mRNA expression in MI compared with normal groups in the test set. **(E)** FOS mRNA expression in MI compared with normal groups in the validation set. An AUC value between 0.8 and 0.9 was considered good, and exceptional when the AUC value was >0.9. ROC, receiver operating characteristic; AUC, area under the ROC curve; MI, myocardial infarction; WGCNA, weighted gene co-expression network analysis.

A Venn diagram was employed to show the intersection of the results of DEGs, PPI network, and WGCNA (Figure 6A). FOS was identified as the sole hub marker (Supplementary Table S2). The ROC curves showed an AUC of 0.833 for FOS in the dataset GSE66360 (Figure 6B) and an AUC of 0.845 for FOS in the validation dataset GSE60993 (Figure 6C), which indicated good efficacy of FOS in the diagnosis of MI. Moreover, the trend of FOS expression was consistent in both datasets (Figures 6D,E). In both, the expression level of FOS in MI patients was higher than that in the control group, and the differences between the groups were statistically significant (*p* < 0.001 in GSE66360, *p* = 0.01 in GSE60993).

### 3.5 Molecular docking analysis

**Figure 7:**
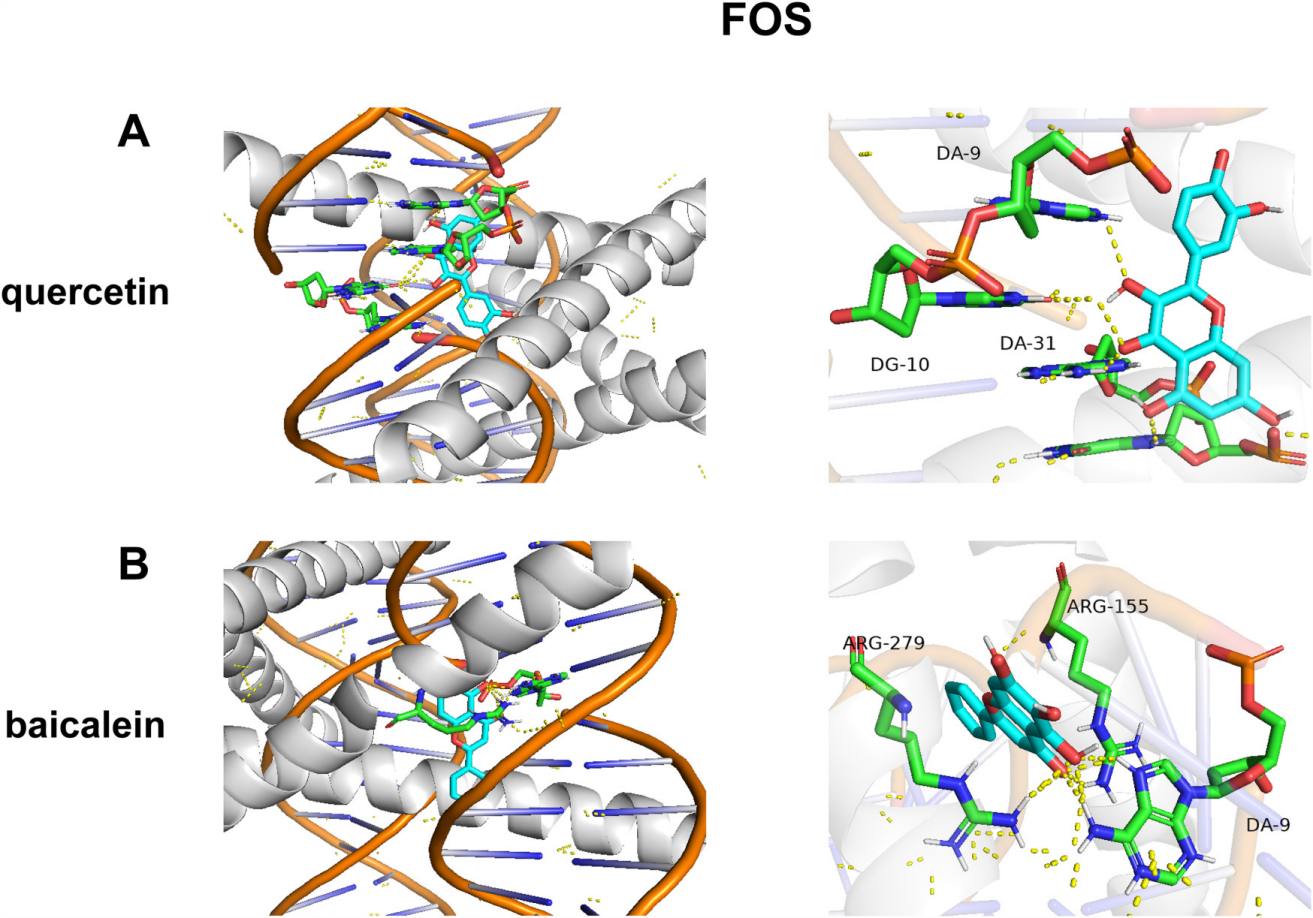
Molecular docking of DYX components and FOS. **(A)** The action mode of FOS with quercetin. **(B)** The action mode of FOS with baicalein.

As shown in Figure 7, quercetin demonstrated the strongest binding affinity (*Δ*G = −10.4 kcal/mol), forming hydrogen bonds with DA-9, DG-10, and DA-31 (Figure 7A). This triple-point interaction suggests direct competition with the FOS/JUN dimer for DNA minor groove occupancy. Baicalein exhibited distinct binding at the protein–DNA interface (*Δ*G = −9.6 kcal/mol), engaging Arg279 of JUN and Arg155 of FOS (Figure 7B). Notably, its flavone scaffold simultaneously intercalates with DA-9, creating a “bridge” between protein and DNA domains (Figure 7B).

### 3.6 Results of immune cell infiltration and correlation with hub biomarkers

**Figure 8:**
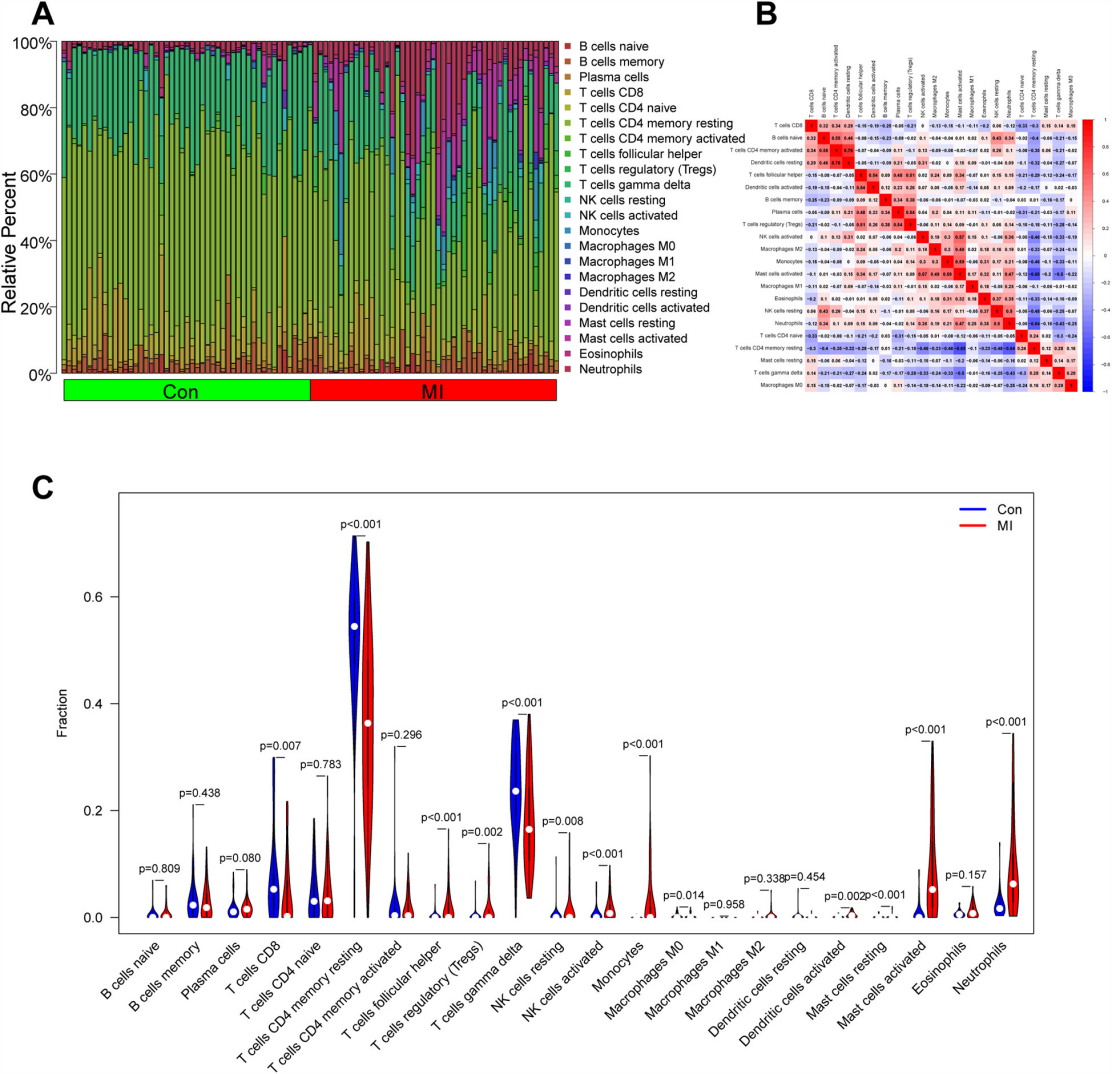
Evaluation of immune cell infiltration. **(A)** Bar plot of proportions of 22 immune cell types in the control group and MI patients. **(B)** Heatmap of correlation between the 22 immune cell types. **(C)** Violin plot of the proportion of 22 immune cell subsets between healthy people and MI patients. MI, myocardial infarction.

The CIBERSORT algorithm was used to analyze the immune cell infiltration. The proportion of 22 immune cells was shown in a bar plot (Figure 8A; Supplementary Table S3). The proportions of immune cells from the whole blood of healthy people and MI patients displayed distinct and group-bias clustering. Resting CD4^+^ memory T cells showed the largest difference in proportion between MI and the control group. Activated CD4^+^ memory T cells had the strongest positive correlation with resting dendritic cells (*r* = 0.76). Meanwhile, resting CD4^+^ memory T cells had the strongest negative correlation with resting mast cells (*r* = −0.65), as shown in Figure 8B. Thirteen types of immune cells were differentially expressed (Figure 8C). Compared with the control group, MI patients generally contained a higher proportion of follicular helper T cells (*p* < 0.001), regulatory T cells (Tregs) (*p* *=* 0.002), gamma delta T cells (*p* < 0.001), resting natural killer (NK) cells (*p* = 0.008), activated NK cells (*p* < 0.001), monocytes (*p* < 0.001), activated dendritic cells (*p* = 0.002), resting mast cells (*p* < 0.001), activated mast cells (*p* < 0.001), and neutrophils (*p* < 0.001), whereas the proportions of CD8^+^ T cells (*p* = 0.007), resting CD4^+^ memory T cells (*p* < 0.001), and M0 macrophages (*p* = 0.014) were relatively lower.

### 3.7 Correlation of FOS with immune cells

**Figure 9:**
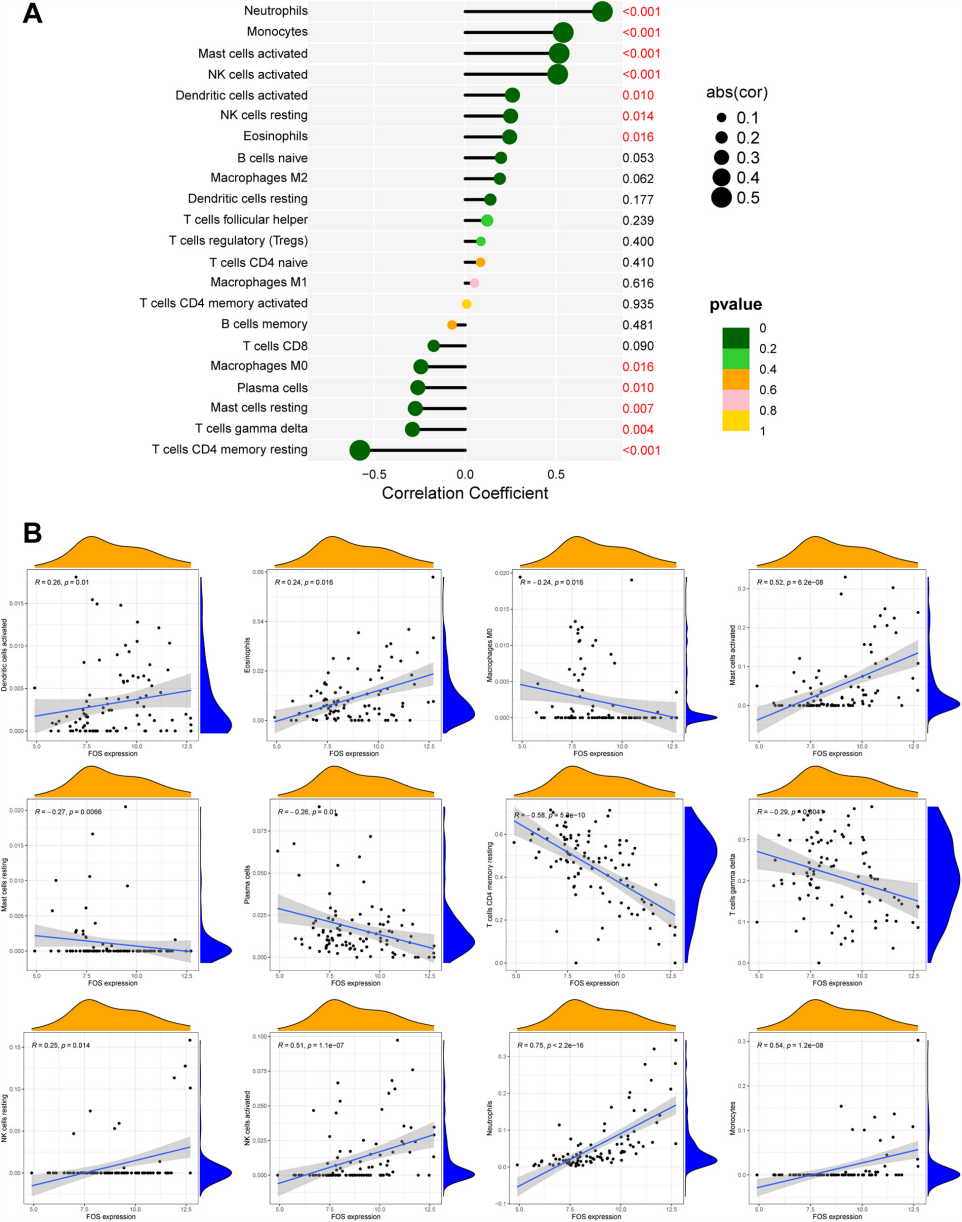
The correlation between FOS and immune cells. **(A)** Forest maps of correlation. **(B)** Scatterplot of correlation. The orange curve shows the density distribution of FOS expression, and the blue curve shows the density distribution of the corresponding immune cell percentage.

Based on the results of correlation analysis, FOS displayed a positive correlation with neutrophils (*p* < 0.001), monocytes (*p* < 0.001), activated mast cells (*p* < 0.001), activated NK cells (*p* < 0.001), dendritic cells (activated) (*p* = 0.010), resting NK cells (*p* = 0.014), and eosinophils (*p* = 0.016) and showed a negative correlation with M0 macrophages (*p* = 0.016), plasma calls (*p* = 0.010), resting mast cells (*p* = 0.007), gamma delta T cells (*p* = 0.004), and resting CD4^+^ memory T cells (*p* < 0.001) (Figures 9A,B). These correlations indicate that FOS may play a significant role in the immune response following MI, influencing the activity and abundance of various immune cell types. The positive correlation with proinflammatory cells such as neutrophils and monocytes suggests that FOS may contribute to their recruitment and activation during the early inflammatory phase post-MI. The negative correlation with certain anti-inflammatory or immune-regulatory cells, such as M0 macrophages and resting mast cells, implies that changes in FOS levels might be associated with the resolution of inflammation and tissue repair.

### 3.8 Single-cell analysis of FOS

**Figure 10:**
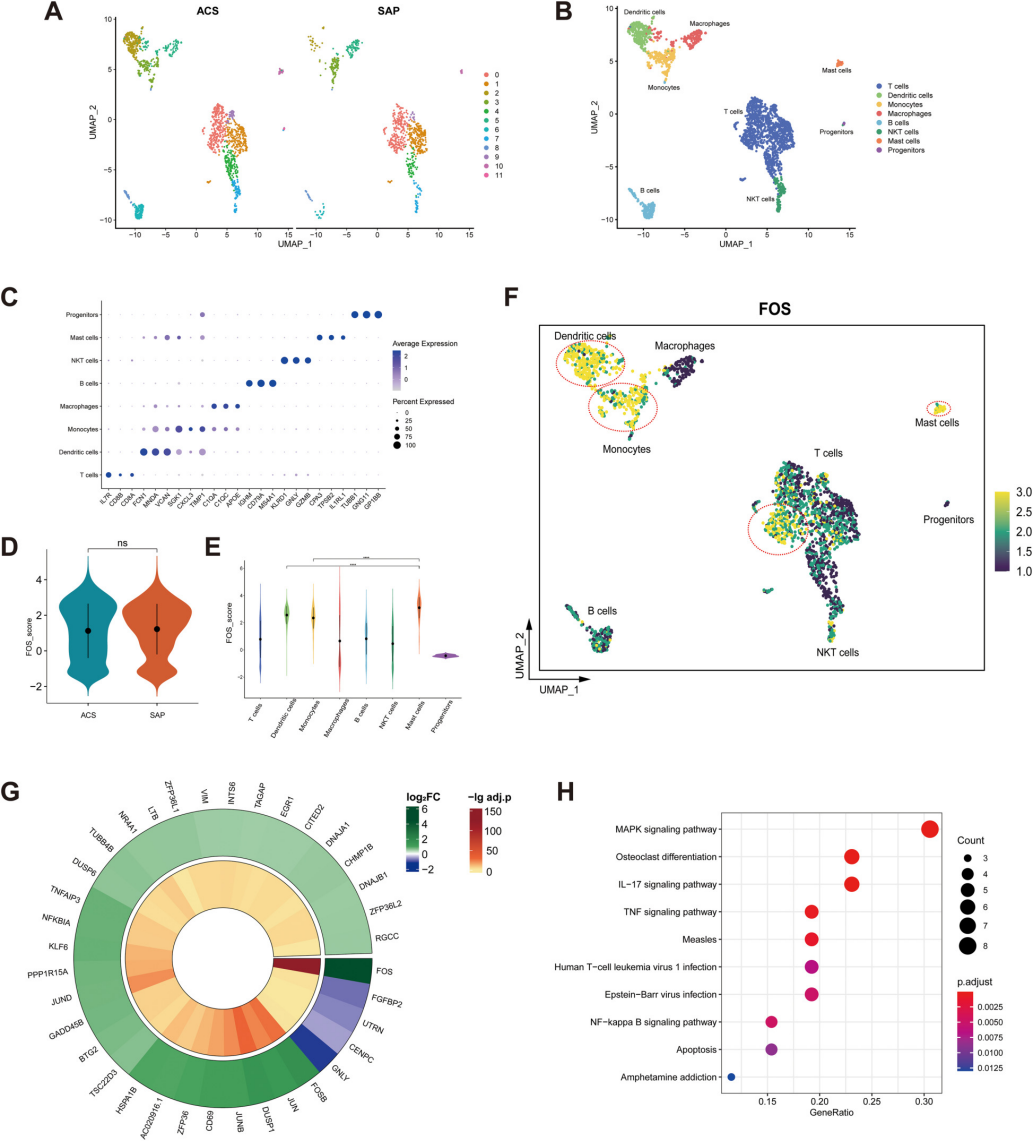
Single-cell analysis of FOS. **(A)** Feature plot of cell clusters in ACS (left) and SAP (right). **(B)** Manual annotation of cell clusters. **(C)** Dot plot of markers. **(D)** Violin plot of FOS expression in ACS and SAP. ns, not significant. **(E)** Violin plot of FOS expression in cell clusters. **** *p* < 0.0001. **(F)** Feature plot of FOS expression in cell clusters. The yellow-colored cells within the red circle indicate higher FOS expression levels. **(G)** Circle heatmap of 36 DEGs between high FOS T cells and low FOS T cells. **(H)** Dot plot illustrating the KEGG enrichment analysis of 36 DEGs. ACS, acute coronary syndrome; SAP, stable angina pectoris; DEGs, differentially expressed genes; KEGG, Kyoto Encyclopedia of Genes and Genomes.

A total of 2,363 cells were obtained after screening. After dimensionality reduction and clustering, these cells were classified into 12 cell clusters (Figure 10A). Cell clusters were manually annotated to eight cell types, including T cells, dendritic cells, monocytes, macrophages, B cells, natural killer T (NKT) cells, mast cells, and progenitors (Figure 10B). Referring to CellMarker, IL7R, CD8A, and CD8B were used as markers for T cells; FCN1, MNDA, and VCAN as markers for dendritic cells; SGK1, CXCL3, and TIMP1 as markers for monocytes; C1QA, C1QC, and APOE as markers for macrophages; IGHM, CD79A, and MS4A1 as markers for B cells; KLRD1, GNLY, and GZMB as markers for NKT cells; CPA3, TPSB2, and IL1RL1 as markers for mast cells; TUBB1, GNG11, and GP1BB as markers for progenitors. The dot plot shows that the markers were highly expressed in the corresponding cell clusters (Figure 10C).

There was no statistical difference in the expression of FOS in stable and unstable plaque samples (Figure 10D). This suggests that FOS is not related to the stability of the plaques. However, given the relatively small sample size of dataset, this remains to be explored in more studies. Among the cell clusters, mast cells had the highest levels of FOS expression, followed by dendritic cells and monocytes (Figure 10E).

In addition, some T cells also showed high levels of FOS expression (Figure 10F). T cells were categorized into high FOS and low FOS groups based on the level of FOS expression. The circle heatmap shows 36 DEGs between groups (Figure 10G). These DEGs were mainly associated with inflammatory signaling pathways such as the MAPK signaling pathway, IL-17 signaling pathway, TNF signaling pathway, and NF-κB signaling pathway (Figure 10H). It suggested that there were subpopulations of T cells that highly expressed FOS and were involved in multiple inflammatory responses in coronary plaques.

## 4 Discussion

Herbal decoctions have been extensively utilized in myocardial infarction (MI) management in China (45, 46). DYX, developed by Professor Tietao Deng, represents a clinically validated formula with demonstrated efficacy not only in MI but also in heart failure, coronary artery disease, and arrhythmia (16). The molecular characterization of DYX's polypharmacology—resulting from its multicomponent nature and multi-target mechanisms—presents unique research challenges (47). To address this, we employed an integrative approach combining network pharmacology and machine learning to decipher DYX-MI interactions while identifying clinically translatable biomarkers.

Our study establishes FOS as the central mediator of DYX's cardioprotective effects. As a core subunit of the AP-1 transcription complex, FOS regulates cellular proliferation and inflammatory responses—processes critical to MI pathogenesis (48, 49). Activation of AP-1 in insulin β-cells is important for the regulation of blood glucose homeostasis (50). In addition to lowering the risk of MI, blood glucose control can also protect cardiomyocytes and reduce the infarct size after a heart attack (51). Animal experiments showed that the expression level of FOS increased with the age of mice and the IL-6 binding site on AP-1 increased (52). It has been shown in an imaging study that aging is associated with the progression of atherosclerosis (53). A study detected increased FOS expression in hypoxic mice cardiomyocytes, and lowering FOS expression could attenuate MI by inhibiting apoptosis (54). Sirtuin3 (SIRT3), a deacetylase, regulates the FOS/AP-1 pathway to alleviate myocardial fibrosis and inflammation (55). The multifactorial association between FOS and MI pathogenesis warrants a mechanistic investigation. Combining immune infiltration profiling with single-cell RNA sequencing, we explored the relationship between FOS and immune cells during the MI process.

Our findings reveal a complex immunomodulatory landscape post-MI, where FOS expression exhibits cell-type–specific correlations with immune infiltration—positively associating with neutrophils, mast cells, dendritic cells, monocytes, and NK cells, while negatively correlating with resting CD4^+^ memory T cells. The diversity of AP-1 regulatory elements makes it possible that FOS expression may have different roles among different immune cells (56, 57).

Neutrophils are among the first immune cells to infiltrate the infarcted myocardium post-MI (58). While their primary role is to clear necrotic tissue (59), excessive neutrophil infiltration and activation can lead to collateral damage to healthy myocardium and exacerbate inflammation (60). FOS expression in neutrophils may play a crucial role in regulating their recruitment and activation. Among the Jun/Fos proteins, only JunD and c-Fos are abundantly expressed in neutrophils, and they are mainly expressed in the cytoplasm (61). This cytoplasmic localization suggests that FOS may regulate neutrophil function through posttranslational modifications or interactions with cytosolic signaling pathways. Mast cells are another key player in the post-MI inflammatory response and may be involved in the entire process of MI (62). They are associated with postinfarction myocardial fibrosis (63). Mast cells exhibit the highest single-cell FOS expression among infiltrating leukocytes. It has been shown that inhibition of FOS expression reduces mast cell activation (64), which could have significant implications for reducing myocardial fibrosis and improving cardiac repair. Specific subpopulations of dendritic cells can activate fibroblasts to participate in cardiac repair after MI but also secrete proinflammatory cytokines to exacerbate inflammation (65). The overexpression of miR-181a, which inhibits FOS expression, has been shown to suppress dendritic cell maturation and attenuate inflammation (66). This suggests that FOS may promote dendritic cell activation and antigen presentation, potentially driving adaptive immune responses and further inflammation. Moreover, p38α in dendritic cells influences IL-12 expression through the MK2-c-FOS pathway, impacting T helper 2 cell differentiation and allergic inflammation (67). Depletion of myocardial resident macrophages after MI allows the recruitment of circulating monocytes (68), and FOS has been shown to play an important role in the differentiation of monocytes to macrophages (69). NK cells cross talk with monocytes and activate each other through the T-β/*γ* interferon/IL-12 signaling pathway (70). However, the specific mechanism of action of NK cells in MI remains to be investigated. It has been suggested that elevated FOS in immune cells such as NK cells in mice may be associated with elevated levels of inflammation (52). This reciprocal activation between NK cells and monocytes, potentially regulated by FOS, could create a positive feedback loop that amplifies inflammation post-MI.

Overall, these findings suggest that FOS may have a significant impact on cardiac repair and inflammation by regulating the interplay between various immune cells postinfarction. DYX may have a significant impact on improving the prognosis of MI by regulating the expression of FOS and shifting postinfarction inflammation to anti-inflammation.

Existing studies have shown that FOS is associated with some components of DYX. It has been shown that ginsenosides can improve lung inflammation in mice by inhibiting FOS expression (71). Trilinolein, isolated from *Panax notoginseng*, inhibits FOS expression and reduces superoxide production in cardiomyocytes (72). The molecular docking analysis revealed robust binding affinities between key components of DYX (quercetin and baicalein) and the FOS protein. Baicalein can reduce reactive oxygen species (ROS) production and protect against ischemia–reperfusion injury in cardiomyocytes (73). Quercetin has been proven to inhibit LDL oxidation and reduce inflammatory levels, thereby slowing down atherosclerosis (74). However, molecular docking primarily predicts binding potential, and experimental validation is required to confirm these interactions. Furthermore, as DYX is a multicomponent formula, synergistic or antagonistic effects among its constituents on FOS activity warrant exploration through systems pharmacology approaches.

While our study demonstrates significant upregulation of FOS in MI patients and robust diagnostic performance (AUC > 0.8 in ROC analysis), its strong correlation with immune infiltration raises valid concerns about specificity as a standalone diagnostic biomarker. Key limitations include its broad involvement in inflammatory responses and unclear temporal dynamics. However, integrating FOS with other emerging biomarkers, such as miRNAs, may address these limitations. FOS—while lacking the ultra-early diagnostic window of miRNAs—may complement these markers by reflecting postinfarction immune dynamics. A combinatorial approach leveraging miRNAs for early diagnosis and FOS for risk stratification or prognosis monitoring might synergistically improve clinical decision-making.

Several limitations of this study should be acknowledged. First, the MI cohort was restricted by a relatively small sample size and single-center recruitment, which may compromise the statistical power and generalizability of our findings. Future multicenter studies with larger and ethnically diverse cohorts are warranted to validate these results. Second, although bioinformatics analyses revealed FOS-associated immune infiltration patterns, the retrospective design precluded validation of causal relationships between FOS expression and immune cell dynamics. Third, while single-cell RNA sequencing highlighted FOS enrichment in mast cells, dendritic cells, and T-cell subsets, the small plaque sample size hinders mechanistic interpretations. Future research should employ CRISPR/Cas9-mediated FOS-knockout in human mast cells, monocytes, and myeloid-specific FOS-knockout mice to verify the mechanisms.

## 5 Conclusion

In this study, our combined network pharmacology and bioinformatics screening identified FOS as a target of DYX for the treatment of MI, with the potential to be a prophylactic and therapeutic biomarker for MI. FOS correlated with neutrophils, activated mast cells, activated dendritic cells, monocytes, NK cells, and resting CD4^+^ memory T cells after MI. This may shed light on the study of FOS as an immunotherapeutic target for MI.

## Data Availability

The original data analyzed in this study are publicly available in the Gene Expression Omnibus (GEO) database. The data can be found at https://www.ncbi.nlm.nih.gov/geo/ under accession numbers: GSE66360, GSE60993, and GSE184073.

## Supplementary material

Supplementary Table 1 Information on the samples in the dataset.

Supplementary Table 2 The intersection among DEGs, WGCNA, and PPI network.

Supplementary Table 3 Results of immune cell infiltration.

## References

[1] Driessen HE van Veen T Boink G. Emerging molecular therapies targeting myocardial infarction-related arrhythmias. Europace. (2017) 19:518–28. 10.1093/europace/euw198 28431070

[2] Li S Peng Y Wang X Qian Y Xiang P Wade SW Cardiovascular events and death after myocardial infarction or ischemic stroke in an older Medicare population. Clin Cardiol. (2019) 42:391–9. 10.1002/clc.23160 30697776 PMC6712383

[3] Qureshi WT Zhang ZM Chang PP Rosamond WD Kitzman DW Wagenknecht LE Silent myocardial infarction and long-term risk of heart failure: the ARIC study. J Am Coll Cardiol. (2018) 71:1–8. 10.1016/j.jacc.2017.10.071 29301615 PMC5757248

[4] Solberg CT Norheim OF Barra M. The disvalue of death in the global burden of disease. J Med Ethics. (2018) 44:192–8. 10.1136/medethics-2017-104365 29079556 PMC5869483

[5] Chatterjee NA Levy WC. Sudden cardiac death after myocardial infarction. Eur J Heart Fail. (2020) 22:856–8. 10.1002/ejhf.1744 31975546

[6] Shah AH Puri R Kalra A. Management of cardiogenic shock complicating acute myocardial infarction: a review. Clin Cardiol. (2019) 42:484–93. 10.1002/clc.23168 30815887 PMC6712338

[7] Braunwald E. Unstable angina and non-ST elevation myocardial infarction. Am J Resp Crit Care. (2012) 185:924–32. 10.1164/rccm.201109-1745CI 22205565

[8] Clarkson SA Heindl B Cai A Beasley M Dillon C Limdi N Outcomes of individuals with and without heart failure presenting with acute coronary syndrome. Am J Cardiol. (2021) 148:1–7. 10.1016/j.amjcard.2021.02.027 33667441 PMC8113093

[9] Laudani C Greco A Occhipinti G Ingala S Calderone D Scalia L Short duration of DAPT versus de-escalation after percutaneous coronary intervention for acute coronary syndromes. JACC-Cardiovasc Inte. (2022) 15:268–77. 10.1016/j.jcin.2021.11.028 35144783

[10] Saito Y Oyama K Tsujita K Yasuda S Kobayashi Y. Treatment strategies of acute myocardial infarction: updates on revascularization, pharmacological therapy, and beyond. J Cardiol. (2023) 81:168–78. 10.1016/j.jjcc.2022.07.003 35882613

[11] Oerlemans MI Mosterd A Dekker MS de Vrey EA van Mil A Pasterkamp G Early assessment of acute coronary syndromes in the emergency department: the potential diagnostic value of circulating microRNAs. EMBO Mol Med. (2012) 4:1176–85. 10.1002/emmm.201201749 23023917 PMC3494874

[12] Tanase DM Gosav EM Ouatu A Badescu MC Dima N Ganceanu-Rusu AR Current knowledge of microRNAs (miRNAs) in acute coronary syndrome (ACS): ST-elevation myocardial infarction (STEMI). Life-Basel. (2021) 11:1057. 10.3390/life11101057 34685428 PMC8541211

[13] Devaux Y Mueller M Haaf P Goretti E Twerenbold R Zangrando J Diagnostic and prognostic value of circulating microRNAs in patients with acute chest pain. J Intern Med. (2015) 277:260–71. 10.1111/joim.12183 24345063

[14] Nissen PH Pedersen OB. Unlocking the potential of microRNA expression: biomarkers for platelet reactivity and coronary artery disease. Semin Thromb Hemost. (2025). 10.1055/s-0045-1805041 40074010

[15] Ding S Zhang H Chen X Xu H. Exploring the prescription patterns of traditional Chinese medicine masters in treating heart failure based on the ancient and modern medical case cloud platform. Chin J Integr Med Cardio-Cerebrovasc Dis. (2024) 23:4244–50. 10.12102/j.issn.1672-1349.2024.23.003

[16] Jin Z Wu W Pi J. Experience of professor Deng Tietao, a master of traditional Chinese medicine, in the treatment of heart failure. Zhongguo Zhong Xi Yi Jie He Za Zhi. (2020) 6:754–5. 10.7661/j.cjim.20190807.331

[17] Mohammadi H Hadi A Kord-Varkaneh H Arab A Afshari M Ferguson A Effects of ginseng supplementation on selected markers of inflammation: a systematic review and meta-analysis. Phytother Res. (2019) 33:1991–2001. 10.1002/ptr.6399 31161680

[18] Chen P Gao Z Guo M Pan D Zhang H Du J Efficacy and safety of *Panax notoginseng* saponin injection in the treatment of acute myocardial infarction: a systematic review and meta-analysis of randomized controlled trials. Front Pharmacol. (2024) 15:1353662. 10.3389/fphar.2024.1353662 38576488 PMC10991745

[19] Wang D Lv L Xu Y Jiang K Chen F Qian J Cardioprotection of *Panax notoginseng* saponins against acute myocardial infarction and heart failure through inducing autophagy. Biomed Pharmacother. (2021) 136:111287. 10.1016/j.biopha.2021.111287 33485065

[20] Xu X Wu Q Pei K Zhang M Mao C Zhong X Ginsenoside Rg1 reduces cardiac inflammation against myocardial ischemia/reperfusion injury by inhibiting macrophage polarization. J Ginseng Res. (2024) 48:570–80. 10.1016/j.jgr.2024.07.003 39583164 PMC11583468

[21] Wang S Zhang Z Lin X Xu DS Feng Y Ding K. A polysaccharide, MDG-1, induces S1P1 and bFGF expression and augments survival and angiogenesis in the ischemic heart. Glycobiology. (2010) 20:473–84. 10.1093/glycob/cwp199 20008963

[22] Li W Ji L Tian J Tang W Shan X Zhao P Ophiopogonin D alleviates diabetic myocardial injuries by regulating mitochondrial dynamics. J Ethnopharmacol. (2021) 271:113853. 10.1016/j.jep.2021.113853 33485986

[23] Zeng JJ Shi HQ Ren FF Zhao XS Chen QY Wang DJ Notoginsenoside R1 protects against myocardial ischemia/reperfusion injury in mice via suppressing TAK1-JNK/p38 signaling. Acta Pharmacol Sin. (2023) 44:1366–79. 10.1038/s41401-023-01057-y 36721009 PMC10310839

[24] Anzai A Anzai T Nagai S Maekawa Y Naito K Kaneko H Regulatory role of dendritic cells in postinfarction healing and left ventricular remodeling. Circulation. (2012) 125:1234–45. 10.1161/CIRCULATIONAHA.111.052126 22308302

[25] Nahrendorf M Pittet MJ Swirski FK. Monocytes: protagonists of infarct inflammation and repair after myocardial infarction. Circulation. (2010) 121:2437–45. 10.1161/CIRCULATIONAHA.109.916346 20530020 PMC2892474

[26] Yan X Anzai A Katsumata Y Matsuhashi T Ito K Endo J Temporal dynamics of cardiac immune cell accumulation following acute myocardial infarction. J Mol Cell Cardiol. (2013) 62:24–35. 10.1016/j.yjmcc.2013.04.023 23644221

[27] Fan Q Tao R Zhang H Xie H Lu L Wang T Dectin-1 contributes to myocardial ischemia/reperfusion injury by regulating macrophage polarization and neutrophil infiltration. Circulation. (2019) 139:663–78. 10.1161/CIRCULATIONAHA.118.036044 30586706

[28] Li J Song Y Jin JY Li GH Guo YZ Yi HY CD226 Deletion improves post-infarction healing via modulating macrophage polarization in mice. Theranostics. (2020) 10:2422–35. 10.7150/thno.37106 32104514 PMC7019150

[29] Zhang J Zhang Y Xin S Wu M Zhang Y Sun L. CXCR7 Suppression modulates macrophage phenotype and function to ameliorate post-myocardial infarction injury. Inflamm Res. (2020) 69:523–32. 10.1007/s00011-020-01335-z 32170348

[30] de Couto G. Macrophages in cardiac repair: environmental cues and therapeutic strategies. Exp Mol Med. (2019) 51:1–10. 10.1038/s12276-019-0269-4 31857583 PMC6923399

[31] Frangogiannis NG. Transforming growth factor-beta in myocardial disease. Nat Rev Cardiol. (2022) 19:435–55. 10.1038/s41569-021-00646-w 34983937

[32] Honold L Nahrendorf M. Resident and monocyte-derived macrophages in cardiovascular disease. Circ Res. (2018) 122:113–27. 10.1161/CIRCRESAHA.117.311071 29301844 PMC5777215

[33] Cui J Pan X Duan X Ke L Song X Zhang W Ophiopogon polysaccharide liposome regulated the immune activity of Kupffer cell through miR-4796 INT. J Mol Sci. (2022) 23:14659. 10.3390/ijms232314659 PMC9735683 36498983

[34] Li XM Yuan DY Liu YH Zhu L Qin HK Yang YB *Panax notoginseng* saponins prevent colitis-associated colorectal cancer via inhibition IDO1 mediated immune regulation. Chin J Nat Medicines. (2022) 20:258–69. 10.1016/S1875-5364(22)60179-1 35487596

[35] Shen T Wang G You L Zhang L Ren H Hu W Polysaccharide from wheat bran induces cytokine expression via the toll-like receptor 4-mediated p38 MAPK signaling pathway and prevents cyclophosphamide-induced immunosuppression in mice. Food Nutr Res. (2017) 61:1344523. 10.1080/16546628.2017.1344523 28747866 PMC5510218

[36] Um Y Eo HJ Kim HJ Kim K Jeon KS Jeong JB. Wild simulated ginseng activates mouse macrophage, RAW264.7 cells through TRL2/4-dependent activation of MAPK, NF-kappaB and PI3K/AKT pathways. J Ethnopharmacol. (2020) 263:113218. 10.1016/j.jep.2020.113218 32755650

[37] Li L Lu Y Liu Y Wang D Duan L Cheng S Network pharmacology analysis of Huangqi Jianzhong Tang targets in gastric cancer. Front Pharmacol. (2022) 13:882147. 10.3389/fphar.2022.882147 35462892 PMC9024123

[38] Wang Y Yuan Y Wang W He Y Zhong H Zhou X Mechanisms underlying the therapeutic effects of Qingfeiyin in treating acute lung injury based on GEO datasets, network pharmacology and molecular docking. Comput Biol Med. (2022) 145:105454. 10.1016/j.compbiomed.2022.105454 35367781

[39] Muse ED Kramer ER Wang H Barrett P Parviz F Novotny MA A whole blood molecular signature for acute myocardial infarction. Sci Rep-UK. (2017) 7:12268. 10.1038/s41598-017-12166-0 PMC5612952 28947747

[40] Park HJ Noh JH Eun JW Koh YS Seo SM Park WS Assessment and diagnostic relevance of novel serum biomarkers for early decision of ST-elevation myocardial infarction. Oncotarget. (2015) 6:12970–83. 10.18632/oncotarget.4001 26025919 PMC4536992

[41] Emoto T Yamamoto H Yamashita T Takaya T Sawada T Takeda S Single-cell RNA sequencing reveals a distinct immune landscape of myeloid cells in coronary culprit plaques causing acute coronary syndrome. Circulation. (2022) 145:1434–6. 10.1161/CIRCULATIONAHA.121.058414 35500048

[42] Stuart T Butler A Hoffman P Hafemeister C Papalexi E Mauck WR Comprehensive integration of single-cell data. Cell. (2019) 177:1888–902. 10.1016/j.cell.2019.05.031 31178118 PMC6687398

[43] Korsunsky I Millard N Fan J Slowikowski K Zhang F Wei K Fast, sensitive and accurate integration of single-cell data with harmony. Nat Methods. (2019) 16:1289–96. 10.1038/s41592-019-0619-0 31740819 PMC6884693

[44] Hu C Li T Xu Y Zhang X Li F Bai J Cellmarker 2.0: an updated database of manually curated cell markers in human/mouse and web tools based on scRNA-seq data. Nucleic Acids Res. (2023) 51:D870–6. 10.1093/nar/gkac947 36300619 PMC9825416

[45] Hao P Jiang F Cheng J Ma L Zhang Y Zhao Y. Traditional Chinese medicine for cardiovascular disease: evidence and potential mechanisms. J Am Coll Cardiol. (2017) 69:2952–66. 10.1016/j.jacc.2017.04.041 28619197

[46] He W Zhao H Xue W Luo Y Yan M Li J Qingre huoxue decoction alleviates atherosclerosis by regulating macrophage polarization through exosomal miR-26a-5p. Drug Des Devel Ther. (2024) 18:6389–411. 10.2147/DDDT.S487476 39749190 PMC11693966

[47] Miao J Gao L Liu X Cai W Chen L Chen M Exploring the therapeutic mechanisms of Yikang decoction in polycystic ovary syndrome: an integration of GEO datasets, network pharmacology, and molecular dynamics simulations. Front Med-Lausanne. (2024) 11:1455964. 10.3389/fmed.2024.1455964 39421869 PMC11484630

[48] Ortins-Pina A Llamas-Velasco M Turpin S Soares-de-Almeida L Filipe P Kutzner H. FOSB Immunoreactivity in endothelia of epithelioid hemangioma (angiolymphoid hyperplasia with eosinophilia). J Cutan Pathol. (2018) 45:395–402. 10.1111/cup.13141 29527734

[49] Shaulian E Karin M. AP-1 in cell proliferation and survival. Oncogene. (2001) 20:2390–400. 10.1038/sj.onc.1204383 11402335

[50] Backes TM Langfermann DS Lesch A Rossler OG Laschke MW Vinson C Regulation and function of AP-1 in insulinoma cells and pancreatic beta-cells. Biochem Pharmacol. (2021) 193:114748. 10.1016/j.bcp.2021.114748 34461116

[51] Caturano A Galiero R Pafundi PC Cesaro A Vetrano E Palmiero G Does a strict glycemic control during acute coronary syndrome play a cardioprotective effect? Pathophysiology and clinical evidence. Diabetes Res Clin Pract. (2021) 178:108959. 10.1016/j.diabres.2021.108959 34280467

[52] Karakaslar EO Katiyar N Hasham M Youn A Sharma S Chung CH Transcriptional activation of Jun and Fos members of the AP-1 complex is a conserved signature of immune aging that contributes to inflammaging. Aging Cell. (2023) 22:e13792. 10.1111/acel.13792 36840360 PMC10086525

[53] Lopez-Melgar B Fernandez-Friera L Oliva B Garcia-Ruiz JM Sanchez-Cabo F Bueno H Short-Term progression of multiterritorial subclinical atherosclerosis. J Am Coll Cardiol. (2020) 75:1617–27. 10.1016/j.jacc.2020.02.026 32273027

[54] Lin B Xu J Wang F Wang J Zhao H Feng D. LncRNA XIST promotes myocardial infarction by regulating FOS through targeting miR-101a-3p. Aging (Albany NY). (2020) 12:7232–47. 10.18632/aging.103072 32315985 PMC7202499

[55] Palomer X Roman-Azcona MS Pizarro-Delgado J Planavila A Villarroya F Valenzuela-Alcaraz B SIRT3-mediated Inhibition of FOS through histone H3 deacetylation prevents cardiac fibrosis and inflammation. Signal Transduct Tar. (2020) 5:14. 10.1038/s41392-020-0114-1 PMC7046732 32296036

[56] Haas S Kaina B. c-Fos is involved in the cellular defence against the genotoxic effect of UV radiation. Carcinogenesis. (1995) 16:985–91. 10.1093/carcin/16.5.985 7767997

[57] van IJzendoorn D Forghany Z Liebelt F Vertegaal AC Jochemsen AG Bovee J Functional analyses of a human vascular tumor FOS variant identify a novel degradation mechanism and a link to tumorigenesis. J Biol Chem. (2017) 292:21282–90. 10.1074/jbc.C117.815845 29150442 PMC5766951

[58] Jiang K Hwa J Xiang Y. Novel strategies for targeting neutrophil against myocardial infarction. Pharmacol Res. (2024) 205:107256. 10.1016/j.phrs.2024.107256 38866263

[59] Daseke MN Chalise U Becirovic-Agic M Salomon JD Cook LM Case AJ Neutrophil signaling during myocardial infarction wound repair. Cell Signal. (2021) 77:109816. 10.1016/j.cellsig.2020.109816 33122000 PMC7718402

[60] Prabhu SD. Cytokine-induced modulation of cardiac function. Circ Res. (2004) 95:1140–53. 10.1161/01.RES.0000150734.79804.92 15591236

[61] Cloutier A Ear T Borissevitch O Larivee P McDonald PP. Inflammatory cytokine expression is independent of the c-jun N-terminal kinase/AP-1 signaling cascade in human neutrophils. J Immunol. (2003) 171:3751–61. 10.4049/jimmunol.171.7.3751 14500675

[62] Kupreishvili K Fuijkschot WW Vonk AB Smulders YM Stooker W Van Hinsbergh VW Mast cells are increased in the media of coronary lesions in patients with myocardial infarction and may favor atherosclerotic plaque instability. J Cardiol. (2017) 69:548–54. 10.1016/j.jjcc.2016.04.018 27288329

[63] Legere SA Haidl ID Legare JF Marshall JS. Mast cells in cardiac fibrosis: new insights suggest opportunities for intervention. Front Immunol. (2019) 10:580. 10.3389/fimmu.2019.00580 31001246 PMC6455071

[64] Wang HN Ji K Zhang LN Xie CC Li WY Zhao ZF Inhibition of c-fos expression attenuates IgE-mediated mast cell activation and allergic inflammation by counteracting an inhibitory AP1/Egr1/IL-4 axis. J Transl Med. (2021) 19:261. 10.1186/s12967-021-02932-0 34130714 PMC8207675

[65] Lee JS Jeong SJ Kim S Chalifour L Yun TJ Miah MA Conventional dendritic cells impair recovery after myocardial infarction. J Immunol. (2018) 201:1784–98. 10.4049/jimmunol.1800322 30097529

[66] Zhu J Yao K Guo J Shi H Ma L Wang Q miR-181a and miR-150 regulate dendritic cell immune inflammatory responses and cardiomyocyte apoptosis via targeting JAK1-STAT1/c-fos pathway. J Cell Mol Med. (2017) 21:2884–95. 10.1111/jcmm.13201 28597963 PMC5661264

[67] Han M Ma J Ouyang S Wang Y Zheng T Lu P The kinase p38alpha functions in dendritic cells to regulate Th2-cell differentiation and allergic inflammation. Cell Mol Immunol. (2022) 19:805–19. 10.1038/s41423-022-00873-2 35551270 PMC9243149

[68] Dutta P Nahrendorf M. Monocytes in myocardial infarction. Arterioscl Throm Vas. (2015) 35:1066–70. 10.1161/ATVBAHA.114.304652 PMC4409536 25792449

[69] Liebermann DA Gregory B Hoffman B. AP-1 (Fos/Jun) transcription factors in hematopoietic differentiation and apoptosis. Int J Oncol. (1998) 12:685–700. 10.3892/ijo.12.3.685 9472112

[70] Knorr M Munzel T Wenzel P. Interplay of NK cells and monocytes in vascular inflammation and myocardial infarction. Front Physiol. (2014) 5:295. 10.3389/fphys.2014.00295 25177297 PMC4132269

[71] Choi JH Lee MJ Jang M Kim HJ Lee S Lee SW *Panax ginseng* exerts antidepressant-like effects by suppressing neuroinflammatory response and upregulating nuclear factor erythroid 2 related factor 2 signaling in the amygdala. J Ginseng Res. (2018) 42:107–15. 10.1016/j.jgr.2017.04.012 29348729 PMC5766696

[72] Yang HY Liu JC Chen YL Chen CH Lin H Lin JW Inhibitory effect of trilinolein on endothelin-1-induced c-fos gene expression in cultured neonatal rat cardiomyocytes. N-S Arch Pharmacol. (2005) 372:160–7. 10.1007/s00210-005-0003-8 16184402

[73] Wang IC Lin JH Lee WS Liu CH Lin TY Yang KT. Baicalein and luteolin inhibit ischemia/reperfusion-induced ferroptosis in rat cardiomyocytes. Int J Cardiol. (2023) 375:74–86. 10.1016/j.ijcard.2022.12.018 36513286

[74] Patel RV Mistry BM Shinde SK Syed R Singh V Shin HS. Therapeutic potential of quercetin as a cardiovascular agent. Eur J Med Chem. (2018) 155:889–904. 10.1016/j.ejmech.2018.06.053 29966915

