# Supplementary material for: FOS as a biomarker for myocardial infarction treatment with Deng's Yangxin Decoction: a systems biology-based analysis
Source: Front Cardiovasc Med. 2025 May 30;12:1488684. doi: 10.3389/fcvm.2025.1488684 (PMC12163015; doi:10.3389/fcvm.2025.1488684)
Supplement: Supplementary Table 1 — Information on the samples in the dataset. [file Table1.docx]

**Supplementary Table 1**

| Datasets | Platform | Participants | Species | Tissues |
| --- | --- | --- | --- | --- |
| GSE60993 | GPL6884 | 17 MI and 7 controls | *Homo Sapiens* | Peripheral blood |
| GSE66360 | GPL570 | 21 MI and 22 controls |  | Circulating endothelial cells |
